# The Silk Road Health Project: How Mobility and Migration Status Influence HIV Risks among Male Migrant Workers in Central Asia

**DOI:** 10.1371/journal.pone.0151278

**Published:** 2016-03-11

**Authors:** Nabila El-Bassel, Louisa Gilbert, Stacey A. Shaw, Gaukhar Mergenova, Assel Terlikbayeva, Sholpan Primbetova, Xin Ma, Mingway Chang, Leyla Ismayilova, Tim Hunt, Brooke West, Elwin Wu, Chris Beyrer

**Affiliations:** 1 Columbia University, School of Social Work, New York, New York, United States of America; 2 Global Health Research Center of Central Asia, Columbia University, Almaty, Kazakhstan; 3 School of Social Service Administration, University of Chicago, Chicago, Illinois, United States of America; 4 Department of Medicine University of California San Diego, San Diego, California, United States of America; 5 Johns Hopkins University, Bloomberg School of Public Health, Epidemiology, International Health, Baltimore, Maryland, United States of America; Simon Fraser University, CANADA

## Abstract

**Objectives:**

We examined whether mobility, migrant status, and risk environments are associated with sexually transmitted infections (STIs) and HIV risk behaviors (e.g. sex trading, multiple partners, and unprotected sex).

**Methods:**

We used Respondent Driven Sampling (RDS) to recruit external male migrant market vendors from Kyrgyzstan, Uzbekistan, and Tajikistan as well internal migrant and non-migrant market vendors from Kazakhstan. We conducted multivariate logistic regressions to examine the effects of mobility combined with the interaction between mobility and migration status on STIs and sexual risk behaviors, when controlling for risk environment characteristics.

**Results:**

Mobility was associated with increased risk for biologically-confirmed STIs, sex trading, and unprotected sex among non-migrants, but not among internal or external migrants. Condom use rates were low among all three groups, particularly external migrants. Risk environment factors of low-income status, debt, homelessness, and limited access to medical care were associated with unprotected sex among external migrants.

**Conclusion:**

Study findings underscore the role mobility and risk environments play in shaping HIV/STI risks. They highlight the need to consider mobility in the context of migration status and other risk environment factors in developing effective prevention strategies for this population.

## Introduction

Globally, low- and middle-income countries continue to witness mass internal and external labor migration and mobility[1, 2], which has been associated with risks for HIV and other sexually transmitted infections (STIs)[3]. Research has identified higher rates of STIs and sexual risk behavior among labor migrants compared to non-migrant men[4–8].

Mobility among migrant workers has been found to increase vulnerabilities to HIV and other STIs[9–13]. HIV risk behavior and STIs among mobile migrant workers has also been associated with several social, economic and policy risk environment factors such as unstable housing, poor living conditions, adverse employment situations, poverty, negligible access to health care services, lack of social support, loneliness, strict migration policies and policing[3, 14–17]. Research among populations at risk of acquiring HIV has increasingly highlighted the importance of understanding and addressing risk factors that comprise environmental context rather than individual behavior[18, 19]. However, few studies have disentangled the extent to which the higher HIV/STIs risk found among migrants is associated with greater mobility versus greater likelihood of their exposure to other risk factors in their environment. Moreover, research has yet to examine whether similar mobility patterns also increase risk for HIV/STIs among comparable groups of non-migrants or internal migrants.

The collapse of the former Soviet Union in 1991 caused political, economic, and social changes in all of the Central Asian (CA) countries, leading to unemployment, greater poverty, and increased migration especially in Tajikistan, Kyrgyzstan, and Uzbekistan[20]. During the past two decades, economic expansion in Kazakhstan and a lack of employment opportunities in neighboring countries have made Kazakhstan a major destination for workers from other CA nations, where an estimated one million labor migrants enter Kazakhstan each year[20]. Several matters influence the high flow of migration to Kazakhstan including proximity to visa-free borders with Kyrgyzstan, greater levels of economic disparity, a growing informal work sector, and a lack of effective governance in the CA region[20]. In Tajikistan, over half of the male population migrate annually to find higher paying employment. Other CA countries, such as Kyrgyzstan and Uzbekistan, also have large migrant labor populations[3].

Migrant workers contribute to economic growth and development in Kazakhstan and other countries[21]. The mobility of migrant workers brings individual and collective advantages such as productive economic exchanges and provision of financial support to family members in the region of origin. Conversely, a number of studies around the globe show that mobility among migrant workers has been found to increase vulnerabilities to infectious diseases such as HIV and other sexually transmitted infections (STIs) due to unstable living conditions, exploitative employment structures, separation from family members, and lack of access to health care services[22].

In Kazakhstan, migrants face multiple challenges such as discrimination, police harassment, and a lack of access to services[21, 23, 24]. In addition, a number of labor laws and agreements further stigmatize this population. Despite these challenges, migrant workers are productive and survive within harsh circumstance due to their strengths. Resilience that promotes coping and adaptive abilities is an important protective factor which reduces risk for diseases such as HIV, yet research on protective factors that mitigate HIV risk behaviors is limited among migrant workers globally and absent in Central Asia[25–28]. Migrants who have positive reasons for migrating and frequent mobility, and who find meaning through group networks, work and securing income, and religious affiliation may be less likely to engage in risk behaviors despite risk environment factors[26, 28].

Although Kazakhstan is one of seven countries that has experienced a 25% increase in HIV incidence from 2001–2009[22, 29], there is limited research on migration, mobility and HIV transmission risks in Kazakhstan and other Central Asian countries. Until recently, injection drug use had been the primary mode of HIV transmission in CA. Now, however, more than half of all new infections occur through heterosexual transmission[30, 31]. A study conducted in Almaty, Kazakhstan, among 422 market vendors from Kyrgyzstan, Kazakhstan, and Uzbekistan showed that migrant men engaged in multiple sexual HIV risk behaviors[8]. Mobility was identified as a risk factor for transmission of HIV and STIs, as those who traveled more often were more likely to have multiple sexual partners and to have recently visited a sex worker[8]. However, this study did not examine risk environment factors, nor did it include a comparison group of non-migrant workers.

A recent systematic review on global migration and HIV transmission underscores that existing studies worldwide have employed non-probabilistic samples, qualitative data, or examined a limited range of risk environment factors that drive HIV risk[3]. This systematic review also indicated that while labor migrant populations are difficult to study due to their frequent mobility and often-illegal status, research methodologies and designs are not fully rigorous in ways that would make study comparisons possible. Moreover, most studies tend to give insufficient attention to internal migrants and/or neglect to include non-migrant workers as a comparison group. Furthermore, biological testing of participants for HIV and STIs is rarely incorporated into the studies. Findings from this systematic review underscored the need for more probabilistic sampling, attention to key subgroups of migrants within samples, attention to a broader range of risk environment factors, and collection of biological samples to document the prevalence of HIV and STIs.

This paper addresses some of the methodological shortcomings and research gaps in prior studies by examining the extent to which mobility is associated with HIV risks and STIs among a large respondent-driven sample (RDS) of three groups of male market workers in Kazakhstan (external migrants, internal migrants, non-migrants) after adjusting for potentially confounding migration risk environment factors and considering the interaction between mobility and migration status. We first describe the mobility, socio-demographic, and risk environment characteristics of the three groups of market workers. We also examine the prevalence of biologically confirmed cases of sexually transmitted infections, including HIV, and sexual risk behaviors (having multiple partners, sex trading, and having any unprotected sex). Next we examine associations between mobility (i.e. spending one or more nights outside Almaty in the past 90 days) and HIV/STI risks, after adjusting for socio-demographic characteristics and structural risk environment characteristics among these three groups. We hypothesize that among all three groups, after adjusting for socio-demographic and structural risk environment factors (legal status, income, debt, policing, homelessness, loneliness, social support, access to medical care, alcohol use), mobility is associated with HIV/STI prevalence and high-risk sexual behaviors, including having multiple sex partners, sex trading, and unprotected sex.

## Materials and Methods

### Recruitment using Respondent Driven Sampling (RDS)

The Columbia University Institutional Review Board approved all study procedures. We recruited a sample of male market workers from Barakholka Market in Almaty, Kazakhstan, the largest marketplace in Central Asia with over 30,000 stalls. We used RDS, where potential participants were compensated both for participating and recruiting others from their network, as a recruitment and incentive system to reach hidden populations[32, 33]. RDS posits that as recruitment continues through waves, a proxy representative sample will be obtained which is independent of the initial seeds[32].

Our sample consisted of labor migrants from Kyrgyzstan, Uzbekistan, and Tajikistan, as well as a comparison sample of internal migrant workers from within Kazakhstan and non-migrant market workers from Almaty. We defined non-migrant market workers as Kazakh citizens who have a primary residence within two hours commuting distance of Almaty city, and internal migrants as workers who maintain a primary residence that is two or more hours outside of Almaty city. Workers were classified as internal migrants based on whether their legal permanent residence or “propiska” was two or more hours outside of Almaty City, as non-legal male residents of Almaty, like external migrants, are not allowed to access health care and most social services and have limited access to jobs in the public sector. By selecting those with a permanent residence two or more hours distant, we sought to identify those who maintain a temporary residence in Almaty and frequently travel to their permanent homes to visit family and friends, similar to many external migrants.

We selected a number of participants as “seeds” to initiate recruitment of subjects for the study. Seeds received three coupons to give to potential participants, who, if eligible, were then enrolled in the study and invited to recruit three additional potential participants. Research assistants screened potential participants for eligibility.

### Eligibility

Men were eligible to participate in the study if they (1) received a valid recruitment coupon or were selected as a seed, were (2) aged 18 to 50, (3) a citizen of Kazakhstan, Kyrgyzstan, Tajikistan, or Uzbekistan, (4) employed in the Barakholka Market in Almaty in the past week, (5) fluent in Russian, Kazakh, or Tajik, and (6) were able to adequately provide informed consent. Participants received approximately $1USD for completing the screening assessment. For each eligible participant recruited, the referring participant received approximately $5USD. Participants provided written informed consent to participate in the study.

All eligible participants completed assessments using Audio Computer-Assisted Self-Interview (ACASI) that collected data on socio-demographics, migration and mobility variables, self-reported HIV risk behaviors, and access to health and HIV services. Interviews on average were between 60–90 minutes. We used biological assays to test for HIV, syphilis, gonorrhea, and Chlamydia. Participants received $10USD for completing the assessment and biological testing.

### Sample RDS Characteristics

**Fig 1:**
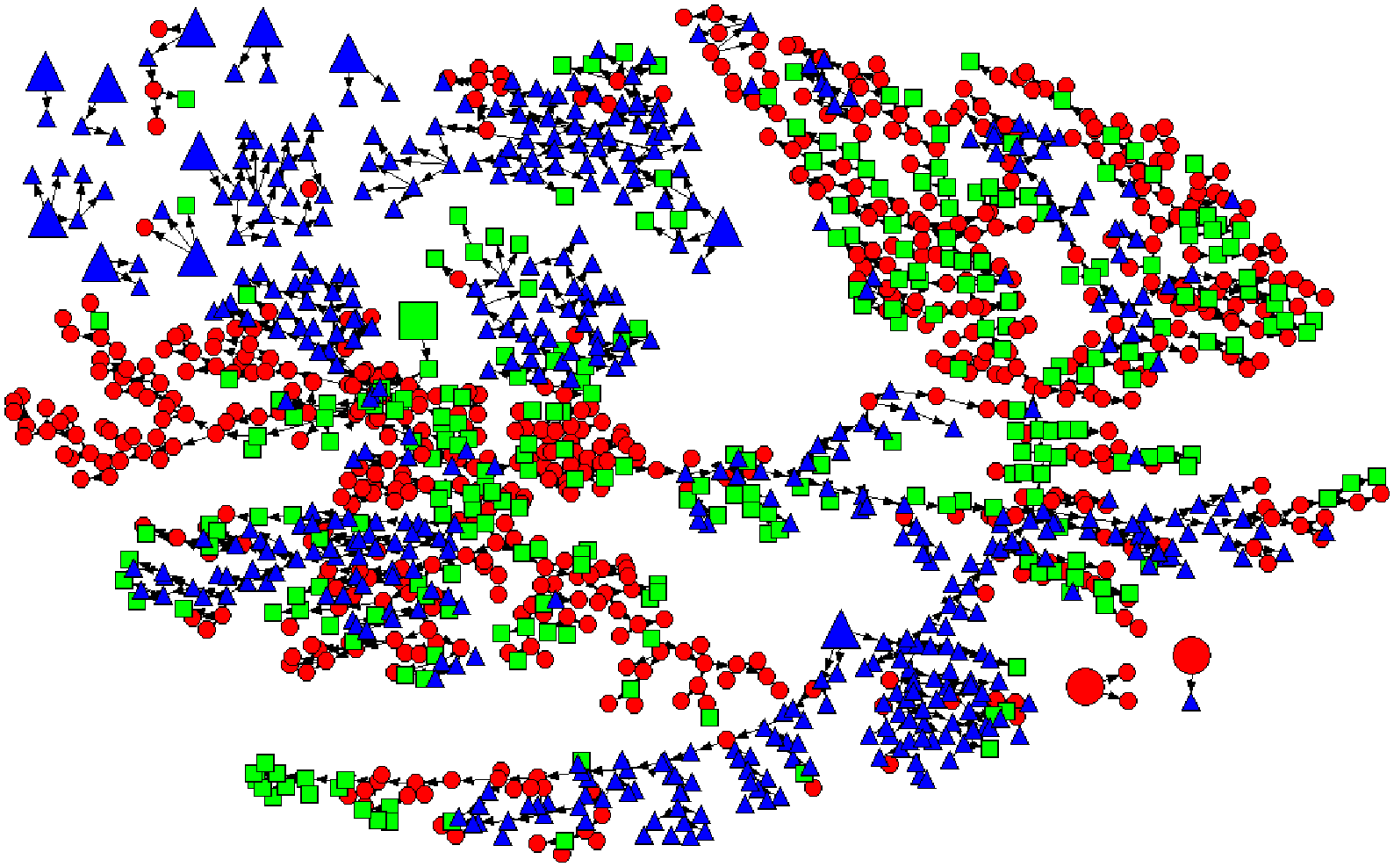
RDS sample from 14 seeds. Red circle = non-migrant, green square = internal migrant, blue triangle-external migrant, large shape = seed.

We selected 14 participants as seeds, including two non-migrants, one internal migrant, and eleven external migrants, to recruit a total sample of 1,342 male labor market workers (See Fig 1). Anyone who met the eligibility criteria could be a seed. We specifically looked for two seeds from each of the five groups (Kazakh internal migrant, Kazakh non-migrant, Kyrgyz migrant, Tajik migrant, and Uzbek migrant). Among 14 isolated recruitment chains, two large chains made up 90% of the sample. The longest chain contained 36 recruitment waves. The sample required six waves to achieve equilibrium and 88% (n = 1,181) of the sample were recruited at the 6^th^ or more waves.

The sample consisted of 562 non-migrant participants (42%), 278 internal migrants (21%), and 502 (37%) external migrants. Network size was measured by the question, “How many people do you know personally who are working at Barakholka (who work at least six months of the year at the marketplace)?” The average network size was 12.93 for the non-migrant group, 12.42 for the internal migrant group, and 32.87 for the external migrant group. The estimated population proportions, after accounting for those who reported larger network sizes, were 0.53 for non-migrants, 0.26 for internal migrants, and 0.21 for external migrants.

We used a homophily index to measure the degree to which respondents in a group recruited from their own group rather than from random mixing[34, 35]. The homophily index measures the degree to which respondents from a particular migrant group recruited others from their own group rather than from random mixing. For example, the homophily index for non-migrants indicates a 28% probability that non-migrants recruited other non-migrants, and a 72% probability that non-migrants recruited others at random, suggesting within-group ties exist as well as a large percentage of random mixing across migrant group[34–36].

### Measures

*Socio-demographic characteristics* included age, education, ethnicity, marital status, living with one’s partner, and having children.

*Employment and living arrangements*: We measured participants’ work responsibilities, income, and living arrangements. Participants were asked about their primary job responsibilities including whether they were an owner, vendor or seller of goods, transporter of goods, or ‘other.’ It was possible for men to say ‘yes’ to more than one primary responsibility. To measure type of residence, we asked participants whether they live in a house/apartment that they own, a house/apartment they rent, their family’s or someone else’s house/apartment, or in another type of residence.

*Migration status*: We defined illegal migrant status as not being a citizen of Kazakhstan and not currently having a permit to work in Kazakhstan.

*Mobility*: We measured mobility by asking participants whether or not they had traveled outside of Almaty in the past 90 days[8]. We selected a 90-day time frame to assess mobility in order to match the 90-day time frame for assessing HIV risk behaviors and as an optimal time frame for recall[37].

*Risk environment*: We examined a number of physical, economic, and risk environment factors as defined by Rhodes[38], including the following: Income level was categorized as whether or not a participant’s income was above or below the living wage in Kazakhstan (identified as 15,999 tenge per month in 2011)[39]. Financial status: Participants were asked whether they sent money home (remittances) to family members who live elsewhere, and whether they were currently in debt. Policing: Participants reported whether they had been questioned by migration police or state officials since working at Baraholka market, and whether they had ever been arrested by migration police or state officials[8]. Food insecurity: We asked whether participants had been without sufficient money for food in the past 90 days. Homelessness: We asked participants whether they had been without a place to sleep in the past 90 days. Perceived loneliness: Participants reported how often they felt lonely. Those who reported loneliness sometimes, often, or almost always were considered to be experiencing loneliness. Social support: The ENRICHD Social Support Instrument (ESSI)[40] evaluated perceived social support. We calculated participant’s average score across the six items. Access to health care: Participants were asked whether or not they had a regular doctor. They also reported whether they had needed to see a doctor in the past 90 days and did not go. Alcohol use: If a participant scored eight or higher on the Alcohol Use Disorders Identification Test (AUDIT), his alcohol consumption was considered to be hazardous[41, 42].

*Sexually transmitted infections*: We assessed for HIV, syphilis, gonorrhea, and Chlamydia through biological assay. Urine specimens were collected from participants and shipped to the Almaty Oblast Skin and Venereal Disease Dispensary to be tested for presence of Chlamydia trachomatis and Neisseria gonorrhea using molecular/DNA amplification assay (BD ProbeTec ET System) with a sensitivity >99.9% and specificity >99.9%. For HIV and syphilis testing, a dried blood spot (DBS) technique was applied. A whole blood spot was obtained by a finger prick and sent to the reference laboratory at the Kazakhstan Republican AIDS Center (RAC). Tests for both biomarkers were conducted using a serial two-test strategy as recommended by the World Health Organization and routinely used at the Kazakhstan RAC. For the serologic surveillance of HIV and syphilis, a standard enzyme-linked immunosorbent assay (ELISA) was used[43]. U.S.-manufactured Abbott Murex Biotech tests were used for the second test. According to the RAC Guidelines for Serological Surveillance, the Murex anti-HIV ABBOTT and the ICE Syphilis Murex ABBOTT each have a reported sensitivity of >99.9% and specificity of 99%. The variable ‘any STI’ was based on whether a participant tested positive for active syphilis (the Murex, VDRL, and TPHA syphilis tests were all positive), gonorrhea, or Chlamydia.

*Sexual risk behaviors* were measured through self-reported data using the Risk Behavior Assessment (RBA), including whether or not the participant was sexually active, had more than one sexual partner in the past 90 days, and whether the participant had exchanged money, goods, or drugs for sex with a woman in the past 90 days. We combined questions on unprotected vaginal or anal sex with a primary female partner, other female partners, or commercial female partners to consider whether the participant had unprotected sex with any female partner in the prior 90 days[44, 45].

### Data Analysis

RDS characteristics and RDS weights were calculated using RDSAT 7.1[46] and then the RDS weights were exported to SAS 9.3. Mean or percentage from the sample and estimating population proportions and the associated 95% confidence intervals for socio-demographic, mobility, and structural risk environment characteristics, as well as sexual risk behaviors were reported for the total sample and by the three migrant groups, including non-migrants, internal migrants, and external migrants. We also examined whether each crude sample mean or percentage fell within the 95% confidence intervals from the population estimates. If the sample mean or percentage was outside the 95% confidence interval, this may indicate that groups were oversampled (beyond the upper limit of 95% confidence intervals) or under-sampled (below the lower limit of 95% confidence intervals) with respect to a variable of interest, either among the total sample or among one of the three migrant groups.

We conducted penalized likelihood logistic regressions[47] to examine the effects of mobility on STIs. The penalized likelihood approach deals with the near-separation situation as the number of STI positive cases was small. We applied multivariate logistic regressions to examine three sexual risk outcome variables, including having more than one sexual partner in the past 90 days, engaging a commercial sex worker in the past 90 days, and having unprotected sex with any partner in the past 90 days. Covariance adjustment for all regressions included age, marital status, income below living wage, remittances, current debt, questioning by migration police or state officials, arrest by migration police or state officials, insufficient money to buy food, homelessness, hazardous drinking, loneliness, perceived social support, lacking a regular doctor, and not seeing a doctor when needed. In order to specify the effects of mobility on the risk outcomes for each migrant group, we included the interaction terms of mobility and migrant group in the regression model, and then combined the main effect of mobility and the associated interaction effects to obtain the estimated effects for each migrant group. RDS individual weights with respect to the migrant groups were also applied to the model. Odds ratios and their 95% confidence intervals are reported. Analyses were performed in SAS 9.3.

## Results

### Socio-demographic, Employment & Living Characteristics

Table 1 presents socio-demographic characteristic results, employment and living arrangements separated by migration status. Average age was 27 (SD = 7.2) (See Table 1). Population estimates suggested Kazakh ethnicity was most common (83.6%) followed by Kyrgyz (5.4%), Karakalpak (3.5%), Uighur (3.6%), Tajik (.9%), Uzbek (.7%), and other (2.3%). An estimated 37.3% of the population was married.

**Table 1:** Socio-demographic characteristics of the sample and population estimates.

	Total (*N* = 1342)		Non-migrants (n = 562)^a^		Internal migrants (n = 278)^a^		External migrants (n = 502)^a^	
	Crude percentage%	Population percentage estimates [95% CIs]	Crude percentage%	Population percentage estimates [95% CIs]	Crude percentage%	Population percentage estimates [95% CIs]	Crude percentage%	Population percentage estimates [95% CIs]
Age (mean)	27.0	26.4 [26.1, 26.8]*	26.8	26.6 [26.0, 27.1]	25.3	24.9 [24.2, 25.6]	28.1	28.0 [27.3, 28.7]
Education (missing 67 cases)								
High school or higher	72.6	76.8 [72.5, 79.6]	74.3	73.0 [67.6, 78.5]	81.6	85.0 [77.8, 89.5]	65.5	76.1 [66.1, 81.2]*
Less than high school	27.5	23.2 [20.4, 27.5]	25.7	27.0 [21.5, 32.4]	18.4	15.0 [10.5, 22.2]	34.5	23.9 [18.8, 33.9]*
Ethnicity								
Kazakh	67.5	83.6 [77.8, 87.7]*	86.3	---	94.6	---	31.3	---
Kyrgyz	5.7	5.4 [2.0, 10.2]	0.2	---	0.0	---	15.2	---
Tajik	15.4	0.9 [0.3, 1.7]*	1.1	---	1.1	---	39.5	---
Uzbek	1.0	0.7 [0.2, 1.3]	0.4	---	0.4	---	2.0	---
Karakalpak	4.2	3.5 [1.6, 5.2]	0.0	---	0.4	---	11.0	---
Uighur	3.1	3.6 [1.9, 6.7]	6.8	---	0.7	---	0.2	---
Other or multiethnic	3.2	2.3 [1.4, 4.1]	5.3	---	2.9	---	1.0	---
Married/common law marriage	43.8	37.3 [32.5, 42.1]*	38.3	32.0 [26.1, 37.5]*	31.7	26.9 [20.2, 36.0]	56.7	53.2 [44.1, 63.1]
Have spouse/girlfriend live together in or near Almaty	26.7	24.0 [20.3, 27.9]	29.0	26.0 [20.8, 30.8]	23.4	20.1 [14.0, 27.7]	25.9	22.3 [15.1, 29.2]
Have children	34.4	29.4 [25.0, 33.6]*	31.0	26.4 [21.1, 32.0]	21.2	16.3 [10.5, 23.1]	45.4	43.1 [33.3, 52.2]
Work at Barakholka Market	65.6	61.0 [56.2, 65.3]*	66.0	65.3 [60.2, 71.0]	60.4	59.7 [50.1, 67.5]	68.1	53.6 [44.7, 64.9]*
As an owner	10.7	4.2 [3.0, 6.1]*	6.2	3.4 [1.8, 5.7]*	5.0	5.2 [2.3, 9.5]	18.9	4.7 [2.6, 10.2]*
As a vendor or sell goods	40.0	30.6 [26.1, 35.2]*	34.9	32.8 [27.7, 39.3]	31.3	31.0 [22.4, 38.9]	50.6	24.9 [18.7, 36.1]*
As a carrier	19.2	23.8 [19.8, 27.9]*	24.0	26.2 [20.8, 32.0]	25.5	24.4 [16.5, 31.4]	10.4	20.8 [11.1, 27.7]*
Other	4.7	6.7 [4.5, 9.0]	5.7	6.9 [3.9, 9.6]	4.7	5.5 [2.3, 9.6]	3.6	8.7 [3.6, 13.7]
Type of residence								
In a house/apt that I own	17.7	16.2 [13.2, 19.3]	23.8	18.0 [13.8, 23.0]*	20.9	20.4 [14.2, 27.8]	9.0	8.6 [4.8, 14.3]
In a house/apt that I rent	73.2	74.5 [70.7, 78.1]	65.7	73.1 [67.5, 78.2]*	68.7	69.3 [61.5, 76.7]	84.0	82.5 [73.3, 87.5]
In family's or someone else's house/apt	7.9	8.4 [6.3, 10.8]	9.3	8.1 [5.3, 11.1]	9.7	10.2 [5.4, 14.9]	5.3	7.0 [3.3, 14.1]
On the street or other	1.3	0.9 [0.4, 1.6]	1.2	0.8 [0.1, 1.6]	0.7	0.2 [0.1, 0.4]*	1.6	1.9 [0.6, 3.8]

* indicates the sample percentage (mean) was outside the 95% confidence intervals of the population percentage (mean).

^a^Non-migrant = Kazakh citizen with a primary residence within two hours commuting distance of Almaty city, Internal migrant = worker who maintain a primary residence two or more hours outside of Almaty, External migrants = labor migrant from Kyrgyzstan, Uzbekistan, or Tajikistan.

Primary employment duties included working within a market stall as a vendor or seller of goods (30.6%), a carrier of goods (23.8%), as a stall owner (4.2%), or in another role (6.7%).

Renting a house or apartment was the most common type of residence (74.5%). Less than one tenth of external migrants owned their residence (8.6%), compared to 20.4% of internal migrants and 18.0% of non-migrants.

### Migration and Mobility

An estimated 33.2% of the population traveled and spent one or more nights outside of Almaty in the previous 90 days (See Table 2). Approximately one fifth of the population (20.3%) had more than one trip during the past 90 days.

**Table 2:** Mobility, structural environment and sexual risks among the sample and estimated for the population.

	Total (N = 1342)		Non-migrants (N = 562)^a^		Internal migrants (N = 278)^a^		External migrants (N = 502)^a^	
	Crude percentage %	Population percentage estimates [95% CIs]	Crude percentage %	Population percentage estimates [95% CIs]	Crude percentage %	Population percentage estimates [95% CIs]	Crude percentage %	Population percentage estimates [95% CIs]
***Migration and mobility***								
Traveled and spent one or more nights outside of Almaty last 90 days	35.5	33.2 [29.1, 37.1]	34.9	34.5 [29.6, 40.3]	43.2	33.5 [26.5, 41.5]*	31.8	31.1 [22.3, 39.6]
# of trips traveled outside of Almaty last 90 days								
one trip	15.0	13.0 [10.4, 16.0]	10.9	11.0 [8.2, 15.0]	15.8	14.6 [8.9, 20.7]	19.2	14.3 [8.6, 21.5]
more than one trip	20.5	20.3 [16.6, 23.6]	24.0	23.6 [19.0, 28.7]	27.3	18.6 [13.8, 24.7]*	12.6	17.3 [10.3, 24.4]
***Structural risk environment***								
Illegal worker in Kazakhstan	---	---	---	---	---	---	65.7	---
Income below living wage	17.1	20.2 [17.0, 23.7]	20.5	20.9 [15.9, 24.6]	17.6	20.2 [13.5, 28.4]	13.0	20.4 [13.1, 27.3]*
Send remittances	62.0	54.5 [50.2, 59.0]*	51.8	51.8 [46.1, 57.7]	57.2	48.6 [40.8, 56.6]*	76.3	64.4 [54.1, 72.4]*
Currently in debt	45.9	37.2 [33.1, 41.5]*	39.5	33.9 [28.9, 39.5]	48.6	41.1 [34.2, 50.5]	51.7	37.3 [30.0, 48.1]*
Questioned by migration police or state officials	42.0	27.5 [24.1, 31.7]*	26.0	20.3 [15.6, 24.6]*	31.3	21.7 [15.4, 28.9]*	66.0	42.6 [34.8, 52.6]*
Ever been arrested by migration police or state officials	35.3	26.0 [22.4, 29.9]*	20.6	18.1 [12.6, 21.2]	19.4	16.6 [11.2, 23.8]	60.6	46.2 [37.2, 54.9]*
Insufficient money to buy food	22.5	28.7 [24.4, 32.4]*	26.2	27.7 [22.5, 32.3]	22.7	26.4 [19.0, 34.9]	18.4	32.5 [23.6, 41.4]*
Homeless	13.5	17.3 [14.1, 21.1]*	14.6	14.6 [10.5, 18.3]	14.0	14.3 [9.1, 20.8]	12.0	24.9 [16.2, 33.5]*
Loneliness	29.0	23.8 [20.2, 27.4]*	20.5	20.4 [16.2, 24.5]	18.7	19.5 [12.9, 26.3]	44.4	31.1 [24.2, 40.6]*
ESSI score (mean)	18.8	17.9 [17.5, 18.3]	18.8	18.3 [17.6, 18.9]	18.4	18.7 [17.8, 19.6]	18.9	16.1 [15.5, 16.7]*
Lack of a regular doctor	87.6	85.8 [82.6, 88.8]	82.7	83.9 [79.9, 87.8]	91.0	89.4 [83.0, 94.5]	91.2	85.2 [79.4, 92.2]
Did not see doctor when needed	18.5	17.1 [13.9, 20.3]	16.4	14.4 [11.3, 18.9]	21.2	19.1 [13.5, 26.6]	19.4	18.1 [11.2, 24.9]
Hazardous drinking	16.4	17.2 [14.0, 20.5]	16.4	17.3 [12.9, 21.6]	15.8	9.5 [6.0, 14.4]*	16.8	23.9 [16.2, 31.5]
***Sexual risks***								
HIV seropositive (missing 5 cases)	0.2	0.0 [0.0, 0.1]*	0.2	---	0.0	---	0.4	---
Syphilis antibody positive (missing 5 cases)	3.3	2.5 [1.5, 3.7]	2.7	2.7 [1.3, 5.0]	2.5	1.4 [0.4, 3.2]	4.4	2.8 [1.1, 5.7]
Any STIs (missing 19 cases)	5.2	6.5 [4.3, 8.9]	6.9	8.8 [5.5, 13.0]	6.2	7.5 [4.0, 11.9]	2.8	2.1 [0.7, 4.8]
Sexually active last 90 days	61.9	60.7 [56.3, 64.8]	67.3	61.8 [56.0, 67.2]*	60.8	63.0 [54.5, 71.8]	56.6	56.4 [46.9, 65.1]
More than one sexual partner last 90 days	28.4	30.1 [26.2, 34.5]	35.4	34.2 [28.5, 39.9]	30.9	34.0 [25.7, 42.5]	19.0	21.4 [14.3, 27.8]
Commercial sex with female partner last 90 days	8.9	8.8 [6.6, 11.4]	8.4	7.6 [4.7, 10.4]	10.1	10.1 [5.4, 16.1]	8.8	11.6 [6.3, 17.6]
Had unprotected vaginal/anal sex with any female partner last 90 days	32.9	28.8 [25.0, 33.2]	28.7	28.6 [23.7, 34.2]	31.7	28.2 [21.9, 37.0]	38.4	28.7 [20.8, 36.7]*

* indicates the sample percentage (mean) was outside the 95% confidence intervals of the population percentage (mean).

^a^Non-migrant = Kazakh citizen with a primary residence within two hours commuting distance of Almaty city, Internal migrant = worker who maintain a primary residence two or more hours outside of Almaty, External migrants = labor migrant from Kyrgyzstan, Uzbekistan, or Tajikistan.

### Risk Environment

#### Financial status

An estimated 20.2% of the target population had an income below the living wage. An estimated half of the population (54.5%) sent remittances home, including 64.4% of external migrants, 48.6% of internal migrants, and 51.8% of non-migrants. Within the population, an estimated 37.2% of men were currently in debt.

#### Legal status and policing

An estimated two thirds of external migrants (65.7%) in the sample were working illegally in Almaty. An estimated 27.5% of the population had ever been questioned by migration or state officials and 26.0% were ever arrested. Population estimates were higher for external migrants, where 42.6% were ever questioned and 46.2% were ever arrested.

#### Food insecurity and homelessness

We estimated that over one-quarter of the population (28.7%) experienced food insecurity and 17.3% experienced homelessness in the past 90 days.

#### Loneliness and perceived social support

Loneliness was experienced by an estimated 23.8% of the population with rates estimated at 31.1% for external migrants, 19.5% for internal migrants, and 20.4% for non-migrants. The average estimated level of perceived social support, as measured by the ESSI, was 18.8 (SD: 7.1), indicating that the majority perceived only limited support to be available.

#### Access to health care

Most of the population (85.8%) did not have access to a regular doctor. These estimates ranged between 83.9% among non-migrants, 89.4% among internal migrants, and 85.2% among external migrants. Additionally, an estimated 17.1% had needed to see a doctor in the past 90 days but did not.

#### Alcohol use

An estimated one fifth of the population (17.2%) engaged in hazardous levels of alcohol consumption.

### Sexual Risks

#### HIV and STIs

Three participants tested positive for HIV (0.2%). A total of 44 participants tested positive for the syphilis antibody (3.3%) and 69 were positive for any STI (5.2%), including gonorrhea, Chlamydia, and active syphilis. In the population, an estimated 2.5% had active syphilis and 6.5% had any STI. An estimated 2.1% of external migrants, 7.5% of internal migrants, and 8.8% of non-migrants were STI-positive.

#### Sexual risk behaviors

Less than two thirds of the population (60.7%) were sexually active in the previous 90 days. Population estimates indicated that 30.1% of men had multiple sexual partners in the prior 90 days, 8.8% purchased commercial sex, and 28.8% had unprotected sex with any female partner. Among external migrants, an estimated 21.4% had multiple partners, compared to 34.0% of internal migrants and 34.2% of non-migrants.

### Relationship between Mobility, STIs, and Sexual HIV Risks

Table 3 presents the odds ratios and their associated 95% confidence intervals from multivariate logistic regressions. The odds ratio associated with the mobility variable for migrant status was obtained by combining the main effect of mobility and the associated interaction effects of mobility and migration status.

**Table 3:** Logistic regression of traveling and risk environment on sexual risks (N = 1342).

		Any STIs, OR [95% CI] (Firth method)	Multiple sexual partners last 90 days, OR [95% CI]	Sex trading last 90 days, OR [95% CI]	Unprotected sex with any female partner last 90 days, OR [95% CI]
***Independent Variables***	***Migrant status***				
Traveled and spent one or more nights outside of Almaty last 90 days	Non migrants	1.82 [1.03, 3.22] *	2.08 [1.44, 2.98] **	2.57 [1.39, 4.73] **	1.58 [1.08, 2.32] *
	Internal migrants	1.62 [0.70, 3.79]	2.41 [1.47, 3.96] **	2.09 [0.97, 4.49]	1.46 [0.86, 2.49]
	External migrants	0.19 [0.02, 1.78]	1.13 [0.59, 2.16]	1.77 [0.77, 4.04]	1.63 [0.90, 2.97]
Age		0.98 [0.94, 1.03]	0.96 [0.93, 0.99] **	0.96 [0.92, 1.01]	1.01 [0.98, 1.03]
married/common law marriage		0.92 [0.50, 1.65]	0.43 [0.29, 0.62] **	0.67 [0.37, 1.19]	3.09 [2.19, 4.38] **
income below living wage		2.58 [1.60, 4.10] **	1.03 [0.75, 1.42]	0.64 [0.37, 1.11]	0.47 [0.32, 0.68] **
send remittances		1.43 [0.92, 2.26]	1.33 [1.02, 1.73] *	1.14 [0.75, 1.74]	0.93 [0.71, 1.22]
currently in debt		1.15 [0.73, 1.82]	1.23 [0.94, 1.61]	1.69 [1.11, 2.56] *	1.65 [1.26, 2.15] **
questioned by migration police or state officials		0.66 [0.34, 1.25]	0.80 [0.57, 1.13]	0.97 [0.59, 1.60]	0.78 [0.55, 1.09]
ever arrested by migration police or state officials		1.07 [0.56, 1.97]	1.10 [0.78, 1.56]	1.47 [0.89, 2.43]	1.10 [0.78, 1.55]
insufficient money to buy food		1.03 [0.61, 1.69]	1.12 [0.82, 1.51]	0.89 [0.55, 1.43]	1.00 [0.73, 1.37]
Homeless		1.02 [0.53, 1.87]	0.54 [0.36, 0.82] **	1.31 [0.73, 2.36]	0.48 [0.32, 0.73] **
Loneliness		0.65 [0.35, 1.15]	0.80 [0.58, 1.09]	0.88 [0.54, 1.45]	1.29 [0.95, 1.75]
ESSI social support score		0.99 [0.96, 1.02]	0.98 [0.97, 1.00]	0.95 [0.93, 0.98] **	1.00 [0.98, 1.02]
lack of a regular doctor		2.28 [1.10, 5.45] *	0.59 [0.42, 0.84] **	0.58 [0.35, 0.96] *	0.87 [0.60, 1.26]
did not see doctor when needed		0.79 [0.40, 1.48]	1.17 [0.83, 1.65]	0.75 [0.44, 1.27]	2.37 [1.69, 3.33] **
hazardous drinking		1.48 [0.82, 2.59]	1.89 [1.32, 2.71] **	2.28 [1.39, 3.76] **	1.46 [1.04, 2.06] *

** p<0.01;

* p<0.05.

#### STIs

For non-migrants, a participant’s mobility was associated with higher odds of having any STI (OR = 1.82 [1.03, 3.22], p<.05), but the same was not true among external migrants (OR = 0.19 [0.02, 1.78]) or internal migrants (OR = 1.62 [0.70, 3.79]). Those who had an income below the living wage (OR = 2.58 [1.60, 4.10], p<.01) or who had no regular doctor (OR = 2.28 [1.10, 5.45], p<.05) were also more likely to have any STI.

#### Multiple sexual partners

Mobility was associated with higher odds of having multiple sexual partners in the last 90 days among non-migrants (OR = 2.08 [1.44, 2.98], p<.01) and internal migrants (OR = 2.41 [1.47, 3.96], p<.01), but not among external migrants. Among the entire population, having multiple sexual partners was associated with younger age (OR = .96 [0.93, 0.99], p<.01), being unmarried (OR = 0.43 [0.29, 0.62], p<.01), and hazardous drinking (OR = 1.89 [1.32, 2.71], p<.01). Higher rates of multiple partners were also associated with some structural indicators of wealth and stability, as those reporting multiple partners were more likely to send remittances (OR = 1.33 [1.02, 1.73], p<.05), not experience homelessness (OR = 0.54 [0.36, 0.82], p<.05), and have a regular doctor (OR = 0.59 [0.42, 0.84], p<.05 for ‘lacking a regular doctor’).

#### Commercial sex

Mobility was associated with purchasing commercial sex in the last 90 days among non-migrants (OR = 2.57 [1.39, 4.73], p<.01) but not among internal migrants or external migrants. Purchasing commercial sex was also associated with being in debt (OR = 1.69 [1.11, 2.56], p<.05), lower levels of social support (OR = 0.95 [0.93, 0.98], p<.01), and hazardous alcohol consumption (OR = 2.28 [1.39, 3.76], p<.01). Alternately, those who were without a regular doctor were less likely to purchase commercial sex (OR = 0.58 [0.35, 0.96], p<.05).

#### Unprotected sex

Recent mobility was associated with unprotected sex with any female partner among non-migrants (OR = 1.58 [1.08, 2.32], p<.05), but not among internal migrants or external migrants. A number of covariates were also significant, where unprotected sex was associated with being married (OR = 3.09 [2.19, 4.38], p<.01), in debt (OR = 1.65 [1.26, 2.15], p<.01), not seeing a doctor when needed (OR = 2.37 [1.69, 3.33], p<.05), and hazardous drinking (OR = 1.46 [1.04, 2.06], p<.05). Having an income below the living wage (OR = 0.47 [0.32, 0.68], p<.01) and experiencing homelessness (OR = 0.48 [0.32, 0.73], p<.01) were associated with lower risk of engaging in unprotected sex.

## Discussion

This study highlights the importance of examining the interaction effects between mobility and migration status on HIV transmission risks among mobile workers, particularly as few studies have utilized interaction effects to elucidate the complex relationships between mobility, migration, and sexual health behaviors. After adjusting for potentially confounding socio-demographic and risk environment characteristics, mobility was associated with increased risks for STIs and a range of HIV risk behaviors for non-migrants, but not for external migrants or internal migrants. These findings contradict those from other studies that suggest external migrants are at higher risk for HIV, where migrants are considered at risk of increasing the transmission of HIV to other populations within receiving countries[4–7]. While our results may be related to unique migration contexts and varying definitions of migration across studies, findings also challenge perceptions or stigma associated with migrants as a source of disease transmission within host countries. We speculate that mobility has a smaller or non-existent association with HIV risk for external migrants because these migrants travel primarily to visit their families rather than to buy goods. Regularly visiting families may reduce or prevent external migrants from purchasing commercial sex or having extra-dyadic affairs because of the social pressure they face when they return to their home countries. Internal migrants, however, usually travel within the country or to a neighboring country to buy goods, but when they extend their travels to countries outside of Central Asia, such as Russia and China, they may also engage in HIV risks. Moreover, our qualitative research with external migrant workers in Kazakhstan[48] has shown that men’s major goals and motivation for migration to Kazakhstan is to send remittances to their families and to fulfill their roles as the major wage earner. They view success in this role as key motivation, which enables them to tolerate harsh living circumstances. This positive motivation and goal-oriented purpose may also play a protective factor and reduce engagement in HIV risks while travelling.

Although the prevalence rate of biologically confirmed HIV was low across all three groups in this population, the high rates of multiple sex partnerships, low rates of condom use across all types of partners in the prior 90 days, and presence of biologically confirmed STIs provide causes for concern. We found a 5% prevalence of gonorrhea, Chlamydia, and syphilis in this sample, but did not test for herpes and HPV, which are more prevalent in this population and also linked with HIV/STI transmission.

This population of mobile workers, particularly non-migrant workers, remains at high risk of becoming infected with HIV if the virus is introduced into their sexual risk networks. Although growing research worldwide has identified how different types of circular mobility patterns are associated with HIV/STI transmission among migrant workers in other epidemic contexts, there remains a dearth of research on how employment-related mobility influences risk for HIV/STIs among non-migrants who need to travel for work, such as market vendors in Central Asia. Recent qualitative research [48] suggests that there is an overlay of commercial sex work opportunities in some Central Asian markets where Kazakhstani vendors travel to buy and sell goods. These vendors may be more likely to purchase commercial sex or exchange goods for sex outside of Kazakhstan as the price is cheaper and they can conceal this behavior from their social circle of family and friends[48].

The study findings suggest several structural risk environment factors associated with migration and mobility may also increase risk for STIs and HIV risks across the three groups of workers. Most notably, earning below minimum wage was associated with increased risk for STIs and having unprotected sex. Being in debt was also associated with having unprotected sex and purchasing sex. Hazardous alcohol consumption was associated with having multiple partners, engaging in commercial sex, having unprotected sex. Lacking a regular doctor was associated with higher rates of STIs. Taken together, these risk environment factors may have played a significant role in fueling sexual risk behaviors among this population of external, internal, and non-migrant male market vendors.

The Baraholka Marketplace in Almaty provides an unique venue to deliver prevention messages through a number of strategies including media campaigns on HIV/STI prevention: opening a health clinic in the marketplace that features HIV/STI prevention along with other health promotion/prevention messages, arranging for a mobile health clinic van to be at the marketplace on a scheduled basis, instituting social networking HIV prevention using peer-led approaches where a few vendors are trained to deliver prevention messages, providing free condoms at the marketplace, and providing free access to confidential HIV/STI testing. All of these strategies must be delivered with an emphasis on protecting the rights and confidentiality of migrants and other market workers. Moreover, in order to ultimately reduce the barriers that put people at risk for HIV, there is a need to improve the general conditions of migrant market vendors, help them sustain their rights, prevent violence against migrants, and reduce marginalization. This can be done through labor agreements, enforcement of rights and responsibilities, a legal registration process, and access to health care and treatment. With the growing role of migration mobility in the HIV epidemic in CA, it may also be effective to address this problem through collaborative global initiatives and policies. There is a need for increased investment in globally focused resources to reduce the vulnerability of people who are affected by migration and HIV[49].

### Limitations

The generalizability of study findings is limited to the Baraholka Market as the sole source of recruitment. Conversely, this study has a number of strengths including a large quasi-representative sample of both external and internal labor migrants and non-migrant laborers recruited through RDS. Additionally, the study uses biological data for HIV and STIs.

## Conclusions

The study findings have important HIV prevention implications, suggesting interventions and policies should be tailored to external, internal, and non-migrant workers, considering their unique set of mobility patterns and the structural factors that shape their risk for HIV/STIs. To date, most HIV prevention programs and policies aimed at key affected populations of mobile workers in Central Asia have largely focused on a curbing the threat of HIV posed by external migrants through mandatory HIV testing and referral of HIV positive migrants to their countries for treatment. This punitive approach often deters labor migrants, who may fear deportment from seeking medical care for HIV, and does not address other key structural risk factors that influence HIV/STI risks. Our findings underscore the urgent need for effective HIV prevention programs and policies targeting the unique needs of internal and non-migrant Kazakhstani residents in mobile professions who remain at high risk of HIV/STIs. This research may help inform the design of programs and policies that more effectively target the unique mobility patterns and structural risk environment factors that shape HIV/STI risks among non-migrants as well as internal and external migrants.
